# “If this is what it means to be old…”: a mixed methods study on the effects of age simulation on views on aging and perceptions of age-related impairments

**DOI:** 10.1007/s10433-023-00793-8

**Published:** 2023-12-06

**Authors:** Laura I. Schmidt, Thomas H. Gerhardy, Leslie Carleton-Schweitzer, Hans-Werner Wahl, Katrin Jekel

**Affiliations:** 1 Institute of Psychology, Heidelberg University, Heidelberg, Germany; 2 Network Aging Research (NAR), Heidelberg University, Heidelberg, Germany; 3 Department of Psychiatry, Psychotherapy and Psychosomatics, AGAPLESION MARKUS HOSPITAL, Frankfurt/Main, Germany

**Keywords:** Age simulation suit, Views on aging, Subjective age, Empathy, Age-related impairments, Sensory impairments

## Abstract

Age simulation suits are a promising tool to increase empathy and to promote positive attitudes toward older adults. However, studies have largely focused on (young) healthcare professionals, are probably biased by social desirability, and have not addressed participants’ views of the aging process triggered by the simulation. The current work combines two studies addressing effects of aging suits on both general and personal views on aging among heterogeneous samples, and exploring spontaneous associations during the simulation. In study 1, *N* = 165 adults (*M* = 37.1 years, *SD* = 15.4, range 18–74 years) answered questionnaires containing general views regarding older adults (“old people are…”) as well as personal perceptions (”aging means to me…”) before and after wearing an aging suit. In study 2, young adults (*N* = 22; *M* = 24.8 years, *SD* = 4.3, range 20–38 years) and middle-aged adults (*N* = 41; *M* = 60.8 years, *SD* = 6.9, range 40–75 years) carried out established geriatric assessments with and without aging suit, and spontaneous impressions on the instant aging experience were recorded. Findings indicated negative shifts in both general and personal views on aging measures in both age groups (*d* = .30 to *d* = .44). Analyses of qualitative data resulted in seven main themes, e.g., “strain/coordination”, “future me”, “empathy/insight”. Group comparisons revealed higher frequencies of future-self related thoughts among middle-aged adults, whereas younger adults mentioned predominantly physical effects of the suit. In conclusion, applying age simulation suits might evoke unintended negative views on aging. In comparison with young adults, middle-aged adults showed broader reflections including thoughts related to emotions, future-self, and potential struggles of older people.

## Introduction

Age simulation suits are increasingly used in a variety of studies and programs to enable younger adults to “walk in the shoes” of older people (Gerhardy et al. 2022). In order to achieve a plausible simulation of old age, the suits aim to imitate a range of age-related functional declines with direct impact on everyday life, for example, hearing and vision impairments, loss of strength, and joint stiffness, by applying restrictors, weights, goggles, ear protection, and gloves. The assumption is that such first-hand experience should enable wearers not only to imagine potential daily challenges of older people but indeed to take the perspective of older adults via a kind of holistic behavior-driven experience. Given the global shift toward aging populations and respective public health and care challenges, simulation suits may gain the status of important tools to educate healthcare professionals or younger adults in general, in order to foster empathy toward older adults.

Research on the effects of aging suits has considerably increased in recent years. In fact, three partly overlapping reviews have been published: Eost-Telling et al. (2020) included 23 studies and focused on empathy and attitudes toward older adults among student samples. Bowden et al. (2021) explored educational effects regarding person-centered care and included 20 studies (9 overlapping). Both reviews concluded that age simulation interventions predominantly resulted in positive effects on knowledge on aging processes, empathy and attitudes toward older adults. However, as study designs and quality of research varied largely, comparisons were difficult. Moreover, almost half of the included papers used multi-mode designs, meaning that an aging suit was just one part of the intervention alongside lectures, workshops, role-plays, or group discussions, making the attribution of effects impossible. The most recent review by Gerhardy et al. (2022) had a broader focus beyond healthcare settings and synthesized 26 age simulation studies (10 overlapping with the previous reviews). One focus was on psychological outcomes, another focus aimed to quantify deteriorations in physical outcomes (i.e., gait, balance) by means of established geriatric assessments and age-relevant reference data. Gerhardy et al. found small-to-medium-sized effects for desired psychological outcomes overall (attitudes: *d*_weighted_ = .33, empathy*: d*_weighted_ = .54); however, particularly high-quality studies did not find meaningful differences between age simulation groups and control groups (Cheng et al. 2020; Lee and Teh 2020). Moreover, some studies even reported negative effects on attitudes toward aging after the simulation (Jeong and Kwon 2020; Jeong et al. 2017; Lucchetti et al. 2017).

### General and personal views on aging: “old people are…” versus “aging means to me…”

Nearly all studies summarized in the mentioned review articles focused on attitudes and empathy toward older adults (as a group) rather than on expectations about one’s own aging process. Referring to the distinction between general and personal views on aging (Wahl and Kornadt 2022), *general* views include socially shared beliefs about aging and how the group of older people is perceived in society (e.g., age stereotypes), whereas *personal* views refer to an individual’s perceptions and experiences with his/her own aging process, that form the image of the aging self (Diehl et al. 2021). The two exceptions also addressing personal views are studies by Henry et al. (2011) among nursing and nutrition science students participating in an aging game (without a standardized suit, but with single components) and by Schmidt et al. (2022) who examined effects on views on aging among middle-aged adults in a pre/post-design with and without an aging suit. Henry et al. (2011) used the Anxiety about Aging Scale (AAS) and found higher feelings of anxiety in some AAS subscales (i.e., fear of losses) after the role-play in the aging game, but also trends toward more positive attitudes that reached significance in the subgroup of nursing students, assessed with the Aging Semantic Differential scale (ASD). Schmidt et al. (2022) reported negative effects after wearing the aging suit on personal views on aging, i.e., expectations of social integration and continuous growth in old age (AgeCog scales; Steverink et al. 2001), higher perceived vulnerability to age-associated impairments (Schwarzer 2001), and higher perceived obsolescence (Brandtstädter and Wentura 1994), whereas general age stereotypes (Kornadt and Rothermund 2011) did not change from pre- to post-assessment.

Hence, emerging findings on personal views on aging seems to point in a reverse direction compared to predominantly positive effects for general views such as attitudes or empathy toward older adults. If additional research would confirm these preliminary findings, it may be that potentially positive general views seen as the result of wearing aging suits may come with the cost of increasingly negative personal views on aging.

### Age simulation against the background of stereotype embodiment theory

The impact of age stereotypes on individual aging processes has been addressed by Becca Levy in her stereotype embodiment theory (Levy 2009), which postulates that individuals internalize positive and negative stereotypical beliefs about aging and older persons from a young age. Since all individuals are constantly exposed to the age stereotypes of their culture, these age stereotypes are reinforced over the years, and as we age, the age stereotypes become increasingly personal and self-relevant. If age simulation suits really trigger negative shifts in personal views, this is problematic, as negative personal views on aging have robustly shown associations with fewer preventive, health-related behaviors (Levy and Myers 2004), lower subjective well-being (Nakamura et al. 2022), cognitive decline (Robertson et al. 2016), lower functional health and even higher mortality (Kotter-Grühn et al. 2009; Sargent-Cox et al. 2012). Hence, there is a need to further explore effects of age simulation interventions on respective aging-related cognitions and perceptions.

Moreover, as previous research has focused on students and young healthcare professionals, middle-aged adults (40 +) or even adults in “third age” between 65 and 80 years (see Baltes and Smith 2003) have been largely overlooked. However, we expect age-heterogeneous samples to be fruitful for several reasons: First, midlife is characterized by first aging experiences and preparations regarding the transition into retirement and old age (Lachman 2015). Second, middle-aged adults might be more sensitive toward age stereotypes and aging-related changes (Kornadt et al. 2017; Miche et al. 2014). Third, the mentioned associations between negative views on aging and consequential adverse developmental outcomes do not apply solely to those in older age, but also to middle-aged adults (Westerhof et al. 2023).

### Effects of simulation of aging: prone to social desirability?

From a methodological standpoint, the common practice of using aging suits in comprehensive lectures and programs, aimed at fostering a deeper understanding of old age, needs to be critically reevaluated when researchers intend to assess the isolated impact of the suits. At present, rather obvious introductions, suggestive items, or even non-anonymous group evaluations outweigh rigorous designs (Gerhardy et al. 2022), and veiled/disguised approaches have not yet been applied. As a consequence of items such as “Has your attitude towards elderly patients changed from playing the game? How?” (Chen et al. 2011, p. 3) or “The program helped me think from perspectives of older adults.” (Han and Kim 2021, p. 136), it remains unclear to what extent wearing the aging suit accounted for effects and what may be attributable to factors such as social desirability within group discussions or interviews, suggestive items or additional teaching about aging processes. This may be especially problematic, when the assessment requires that older adults are to be judged as a group, i.e., if age stereotypes, attitudes, or empathy are targeted, whereas personal views on aging might be less prone to answering in a manner that is generally viewed favorably by others. Hence, not mentioning in advance which effects are expected by applying aging suits and even trying out veiled instructions and/or undirected open questions might clarify effects more closely.

### Novel contribution of the present work and research aims

In this work, we aimed to provide novel insights on the effects of wearing an aging suit by assessing both *general* and *personal* views on aging with standardized questionnaires (study 1) and an open unguided qualitative approach (study 2) among two age-diverse samples. Thereby, we aimed to deepen the understanding regarding cognitions on one’s own aging process, that have hardly been investigated so far in age simulation research, especially among middle-aged adults. In study 1, we expected a negative shift in personal views on aging induced by the aging suit in standardized pre/post-questionnaires, i.e., less positive views and more negative views. We decided not to derive a directional hypothesis for general views, as previous literature is mixed and some studies with more rigorous designs revealed no effects. In study 2, we took advantage of an experimental study on biomechanical effects of age simulation in a motion capturing laboratory environment (Gerhardy et al. 2023). Here, we aimed to investigate effects qualitatively by collecting spontaneous associations while still wearing the suit through an open question approach in order to reduce biases due to social desirability in common age simulation practice.

Following Lachman (2015) and Kornadt et al. (2017), we expected middle-aged adults to be more sensitive to the simulation experience which might relate to a higher frequency of mentioned effects experienced during the simulation.

## Methods

Both studies were approved by the ethics commission of the Faculty of Behavioral and Cultural Sciences at Ruprecht-Karls-University Heidelberg, Germany. All participants provided informed consent. In study 2, participants were offered 20€.

### Recruitment and samples

Study 1: Participants were recruited via convenience sampling at two open door days of the Network Aging Research at Heidelberg, a health fair in Alzey, Rhineland-Palatinate, Germany, and four university classes at Heidelberg University. The final sample consisted of *N* = 165 adults without mobility impairments aged 18 to 74 years (*M* = 37.1, *SD* = 15.4), 69% were women, and 69% had a high school diploma (Abitur).

Study 2: Two samples were approached: Young adults (*N* = 22) were recruited via mail distribution lists at Heidelberg University and snowballing procedures (age: *M* = 24.8 years, *SD* = 4.3, range 20–38 years; 46% women). The younger subsample had a body mass index (BMI) ranging from 18.0 to 27.8 (*M* = 22.0; *SD* = 2.3) and rated their (corrected) eyesight as very good (77%) or good (23%). Middle-aged adults were originally recruited for an age simulation study with a different focus, namely the measurement of physical effects in a motion capturing laboratory. They were contacted via opportunity sampling from a pool of residents of the Rhine-Neckar-Area that regularly participate in studies and screened via telephone for exclusion criteria (i.e., chronic illness, pain or mobility impairments). Participants in this middle-aged to young-old sample (*N* = 41) were 40 to 75 years old (*M* = 60.8 years, *SD* = 6.9; 75% women), had a BMI ranging from 19.5 to 35.4 (*M* = 24.6, *SD* = 3.87), and 24% rated their (corrected) eyesight to be very good, 66% good and 10% satisfactory.

### Aging suit and procedure

**Fig. 1:**
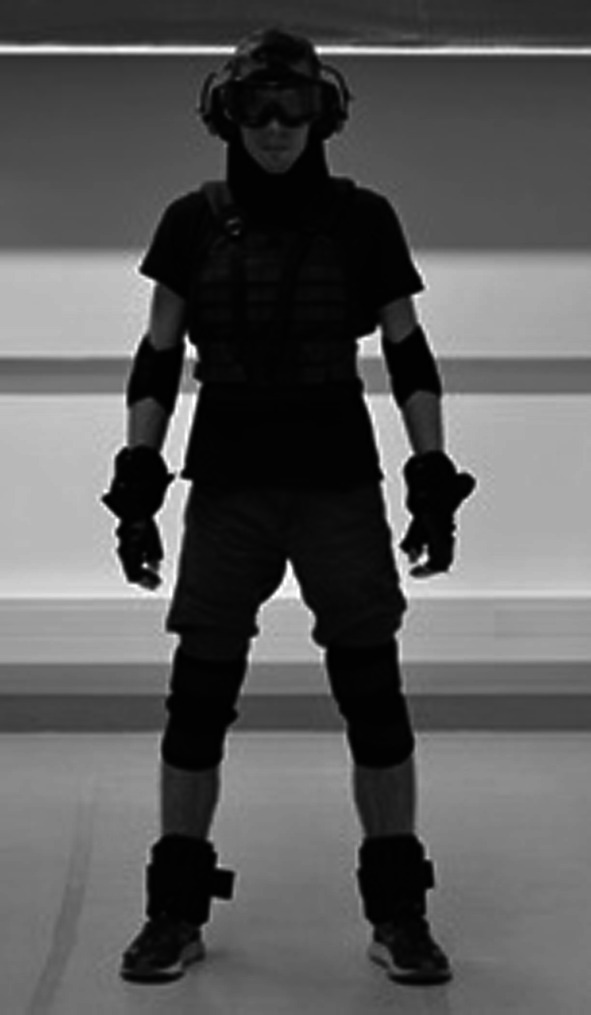
GERT© age simulation suit including goggles, hearing protectors, cervical collar, knee and elbow restrictions, weight vest, and weight cuffs on wrists and ankles

For both studies, we used the age simulation suit GERT (https://www.produktundprojekt.de), which consists of a weight vest, wrist and ankle weights, knee and elbow restrictors, a cervical collar, gloves, hearing protection, goggles, and overshoes (see Fig. 1). In study 1, participants filled out the pre-questionnaires, were then equipped with the suit, and instructed to carry out a defined set of everyday tasks (e.g., climbing stairs, sitting down and standing up, counting an amount of money, tying shoes, reading a bus schedule). After taking off the suit, they filled out the post-questionnaires. In study 2, the age simulation intervention included established geriatric assessments and motion tasks, e.g., the Timed Up and Go test, that were carried out with and without the aging suit in the motion capturing laboratory.

### Measures

#### Background characteristics

As demographic variables, we assessed chronological age, sex, and education (years of schooling) in both studies. In study 2, health-related information was collected in the telephone screening.

#### Views on aging

In both studies, two items were used to measure general subjective age (“How old do you feel?”) and subjective age while wearing the suit (“How old do you feel with the suit?”). The mean difference between subjective age with the suit and general subjective age was calculated to quantify the effects of the suit on felt age (instant aging effect). In study 1, general views on aging were assessed with 12 adjectives (i.e., independent, solitary, inflexible, active, demanding, depressed, passive, balanced, needy, serious, strenuous, and satisfied) following the prompt “many old people are…” on a scale from 0 = strongly disagree to 4 = strongly agree. Two subscales, namely negative views (Cronbach’s *α*_pre_ = .74, *α*_post_ = .81) and positive views (*α*_pre_ = .62, *α*_post_ = .66), were formed.

Personal views on aging were assessed on the same scale with 11 items following the prompt “aging means to me…” (i.e., enrichment, physical decline, fear of the future, acceptance of gains and losses, new contact possibilities, more time, to be able to cultivate hobbies/interests, more quality of life, loss of social contacts, negative experiences outweigh). Two subscales, namely loss-related views (Cronbach’s *α*_pre_ = .56, *α*_post_ = .65) and gain-related views (*α*_pre_ = .67, *α*_post_ = .77), were formed. The selection of items was based on longer established questionnaires assessing general and personal views on aging (Gluth et al. 2010; Kruse and Schmitt 2006).

#### Qualitative data

In study 1, participants were asked to write down their experience in an open text field following the question “which restriction (due to the suit) was most burdensome for you?” after taking off the suit. In study 2, qualitative data were assessed while still wearing the suit through one open question (“What was it like for you to wear this suit?”) and recorded using a smartphone. Participants were asked to speak freely and spontaneously about anything that came to their mind. The interview was carried out by a researcher (TG) who had no relationship with the participants prior to the study.

### Data analysis

Study 1: An a priori power analysis for *t* tests (dependent means) was conducted using G*Power version 3.1.9.7 (Faul et al., 2009). Results indicated that the required sample size to achieve 90% test power (*α* = .05) in order to detect medium-sized effects (Cohen’s *d* = .50) was *N* = 44, and *N* = 265 to detect small effects (*d* = .20). All analyses were performed using SPSS version 27.

Study 2: For the analysis of qualitative data, audio files of the recorded reflections while wearing the suit were transcribed with f4transkript and structured with ATLAS.ti9. Due to missing/broken audio files, two participants of the younger and one of the middle-aged group had to be excluded. Braun and Clarke’s (2006) six-step thematic analysis was applied. To capture the content of the interviews optimally, different approaches were taken. At earlier stages, we searched for prominent topics via word frequencies in ATLAS.ti9. Due to the open answer format, this brought no guidance for analyses. We then tried to map the subthemes found in Bowden et al. (2020) (e.g., “learning through experience”) onto our data as a deductive approach. Eventually, the themes did not match our data sufficiently, leading to an inductive approach. The resulting themes were then compared to themes discovered in study 1 and partly unified leading to seven main themes. Two independent researchers (LIS and LCS) then rated each transcript according to the occurrence of the themes. Afterward, disagreements were discussed and resolved. A sensitivity power analysis for chi-square tests revealed that with the given *N* = 60, a power of 90%, and *α* = .05, only medium-to-large effects can be detected (ω = .42; critical *χ*^2^ = 3.84).

## Results

Table 1 shows age measures with and without the aging suit for both studies. The subjective age with the suit reached scores corresponding to third age in study 1 (*M* = 73.3 years, *SD* = 11.4) and the young sample in study 2 (*M* = 71.1 years, *SD* = 12.9), whereas fourth age (80 +) was reached in the middle-aged sample of study 2 (*M* = 81.1 years, *SD* = 9.6). The instant aging effect, i.e., the difference between general subjective age and age with the suit, showed considerable variance and was lower among middle-aged participants in study 2 (*M* = 29.7 years, *SD* = 13.0) than among their younger counterparts (*M* = 46.2 years, *SD* = 15.5).

**Table 1:** Age measures with and without the age simulation suit (studies 1 and 2)

Variable	Study 1		Study 2, young		Study 2, middle-aged	
	Min–Max	*M* (*SD*)	Min–Max	*M* (*SD*)	Min–Max	*M* (*SD*)
Chronological age	18–74	37.2 (15.4)	20–38	24.8 (4.3)	40–75	60.8 (6.9)
Subjective age^a^	16–72	32.4 (12.5)	20–40	24.6 (4.6)	30–69	51.6 (10.1)
Subjective age suit^b^	30–90	73.3 (11.4)	38–85	71.1 (12.9)	50–100	81.1 (9.6)
Instant aging effect^c^	0–70	41.6 (15.8)	13–65	46.2 (15.5)	2–60	29.7 (13.0)

*N* (study 1) = 165, *N* (study 2, young adults) = 22, *N* (study 2, middle-aged adults) = 41

^a^general subjective age (“How old do you feel?”), ^b^subjective age while wearing the suit (“How old do you feel with the suit?”). ^c^difference score: subjective age with the suit minus general subjective age

### Changes in general and personal views on aging

In study 1, data of *N* = 126 participants with complete pre- and post-questionnaires were analyzed with respect to views on aging measures (see Table 2). Paired comparisons indicated that both general and personal views were significantly affected by the age simulation, with general negative views and personal loss-related views increasing, and general positive views and personal gain-related views decreasing after the simulation. Effect sizes were small to medium. With respect to sociodemographic variables (age, sex, and education), no significant associations emerged for general views. For personal views, we only found a trend for age, with older adults reporting more personal gain-related views (*r* = .14, *p* = .08) before the age simulation, which was more pronounced if subjective age was used (*r* = .17, *p* = .034) instead of chronological age. Moreover, women reported more loss-related views after the simulation than men (*t* = − 2.45, *p* = .016, Cohen’s *d* = − 46). We calculated difference scores for the four views on aging measures to test if the observed changes were associated with sociodemographic variables, which was not the case for age and sex, but for participants with higher education the negative shift in personal losses was smaller (*r* = .20, *p* = .024).

**Table 2:** General and personal views on aging before and after the age simulation (study 1)

	*M*_*pre*_	*SD*_*pre*_	*M*_*post*_	*SD*_*post*_	t	*p*	Cohen’s d [95% CI]
*General views on aging*							
Negative views	2.08	.58	2.27	.58	− 4.37	.000	− .39 [− .57; − .21]
Positive views	2.28	.51	2.13	.54	3.38	.001	.30 [ .12; .48]
*Personal views on aging*							
Loss-related views	2.28	.66	2.43	.65	− 3.05	.003	− .27 [− .45; − .09]
Gain-related views	2.44	.51	2.23	.59	4.96	.000	.44 [ .26; .63]

*N* = 126

General views (“many old people are…”) were assessed on a scale from 0 = strongly disagree to 4 = strongly agree; subscales: negative views and positive views

Personal view on aging (“aging means to me…”) was assessed on a scale from 0 = strongly disagree to 4 = strongly agree; subscales: loss-related views and gain-related views

### Qualitative data: how do participants experience the age simulation?

In study 1, we asked participants to name the most burdensome features of the suit in an open text field. Four categories emerged and were coded by two independent reviewers (LCS and TG): (1) Hearing impairments were named by 43.9% of participants (rater agreement = 99.4%), (2) vision was named by 47.7% (agreement = 100%), (3) strain and limited coordination, i.e., due to weights by 54.2% (agreement = 93.3%), and (4) other associations by 6.5% (agreement = 90.3%). Frequencies were not related to sociodemographic variables (*ps* > 05).

In study 2, following the thematic analysis described above resulted in seven main themes: (1) hearing, (2) vision, (3) strain and coordination, (4) negative affect, (5) future me, (6) empathy and insight, (7) other associations (see Table 3 for additional examples of quotes).

**Table 3:** Additional examples of quotes while wearing the age simulation suit for each theme (study 2)

Description of themes (agreement rates)	Example quotes
Theme 1: hearing (95% agreement)	“Everything was more tiring, duller. Even the movements themselves, even now when you talk, you hear yourself muffled and you perceive yourself completely differently.” (T003)
Theme 2: vision (96.7% agreement)	“To me, the worst feature of this suit is actually the insufficient sensory perception of eyes and ears.” (E4064)
Theme 3: strain and coordination (86.7% agreement)	“To me, the heavy legs were the worst. That you don’t have control over them, (incomp.) [that there] is a distinct gait disturbance.” (E4131)
Theme 4: negative affect (88.3% agreement)	“That does make you a little sad, I have to say, when the visual field is [tinted] yellow.” (T016) “I feel considerably older. That is very distressing.” (E4201)
Theme 5: future me (86.7% agreement)	“You hope that if you get really old that you don’t have these limitations that you experience through this additional equipment.” (T4081) “(…) but I can imagine that this is how it really is in old age. When you can’t do things like you used to, when your legs are heavy or perhaps you’re also in pain and you simply can’t perform the movements anymore.” (T4051)
Theme 6: empathy and insight (93.3% agreement)	“I’m relating all of this a lot to my 93 year old mother in-law who is exactly in this condition. Therefore, this was very frustrating to me. If this is the reality I feel even more sorry for her than I already do.” (E4225) “Then you do realize how the demands you place on somebody who might present themselves as very fit but actually experience these limitations (…) how it can burden them to carry three or two grocery bags home or to go to the train carrying a suitcase, or simple tasks like taking out the trash or going down some stairs.” (T4124)
Theme 7: other associations (86.7% agreement)	“I am a diver and before you go into the water, right this moment that’s what this feels like. You’re so heavy, you’re have all the lead and yes, that’s why I feel like finally going into the water so that it gets lighter.” (T004) “[It] definitely rather felt like sports training as soon as the headphones and (…) the goggles were off.” (T009) “When I think about some people perhaps carrying this weight as bodyweight then I think to myself: Good heavens! Well, I will aim to lose some more kilos myself. Because it really is straining for your body to carry so much weight.” (E4098) “Perhaps you walk like this when you’ve got a certain alcohol level in your blood. It was a little difficult to walk in a straight line” (E4098)

Qualitative data were assessed through one open question (i.e., “What was it like for you to wear this suit?”). Codes were generated by two independent reviewers (LIS and LCS)

Themes 1 (*hearing*), 2 (*vision*), and 3 (*strain and coordination*) summarized any negative physical effect participants reported experiencing while wearing the suit due to hearing protection, goggles, or the weights/restrictions of the GERT. Participants talked about varying physical limitations due to the equipment of the suit: “When I was turning around the cone, I felt that I didn’t really have an overview. Meaning, I can’t really see where I have to go. […] So altogether, I felt clumsier, more insecure.” (E4099). There was no obvious pattern as to which restriction was considered most limiting or straining. It rather seemed that the suit had different negative effects interindividually.

Theme 4, *negative affect,* included negative feelings evoked by the suit as well as mental burdens: “This is not only physical, but I feel psychologically shackled by this heavy weight.” (E4018). Theme 5, *future me,* included any statement participants made about themselves in the future. Wearing the suit made participants picture their own aging, and critically reflect on future situations: “And when I think about this perhaps being my condition in older age, I don’t hope so, but yes, it would indeed be a big burden. Whether it’s grocery shopping or just taking a walk; ultimately, whatever you do is a bigger exertion.” (E4024). One person expressed extremely negative thoughts and emotions when imagining the experienced condition as their future-self. Considering the general context, it is unlikely that this person said the following in all seriousness: “If this is what it’s like to be old I will consider getting enough pills beforehand to commit suicide at some point before I get old. Because this just sucks. If this is what it means to be old.” (E4101).

Theme 6, *empathy and insight,* covered statements that expressed empathy toward older people or any kind of insight into their situation. Wearing the suit made some people think about certain loved ones and helped them putting themselves into their position: “I can more or less imagine how my mother feels at 89: this was also the reason why I did this here. Because I wanted to experience this, having difficulty doing anything and I do feel this way.” (T4063). Additionally, the experience helped some participants to critically reflect on their day-to-day perception and expectations of older people.

Apart from reports concerning direct effects of the suit and aging-related topics, Theme 7 included *other associations* evoked. Some people spontaneously compared the effects of the suit to certain sports or considered the intervention a substitute for their (usual) training. In contrast, other participants stated feelings of obesity, which even motivated one participant to lose weight, or compared the simulation to the influence of alcohol (see Table 3).

#### Frequencies and age group comparisons (study 2)

**Fig. 2:**
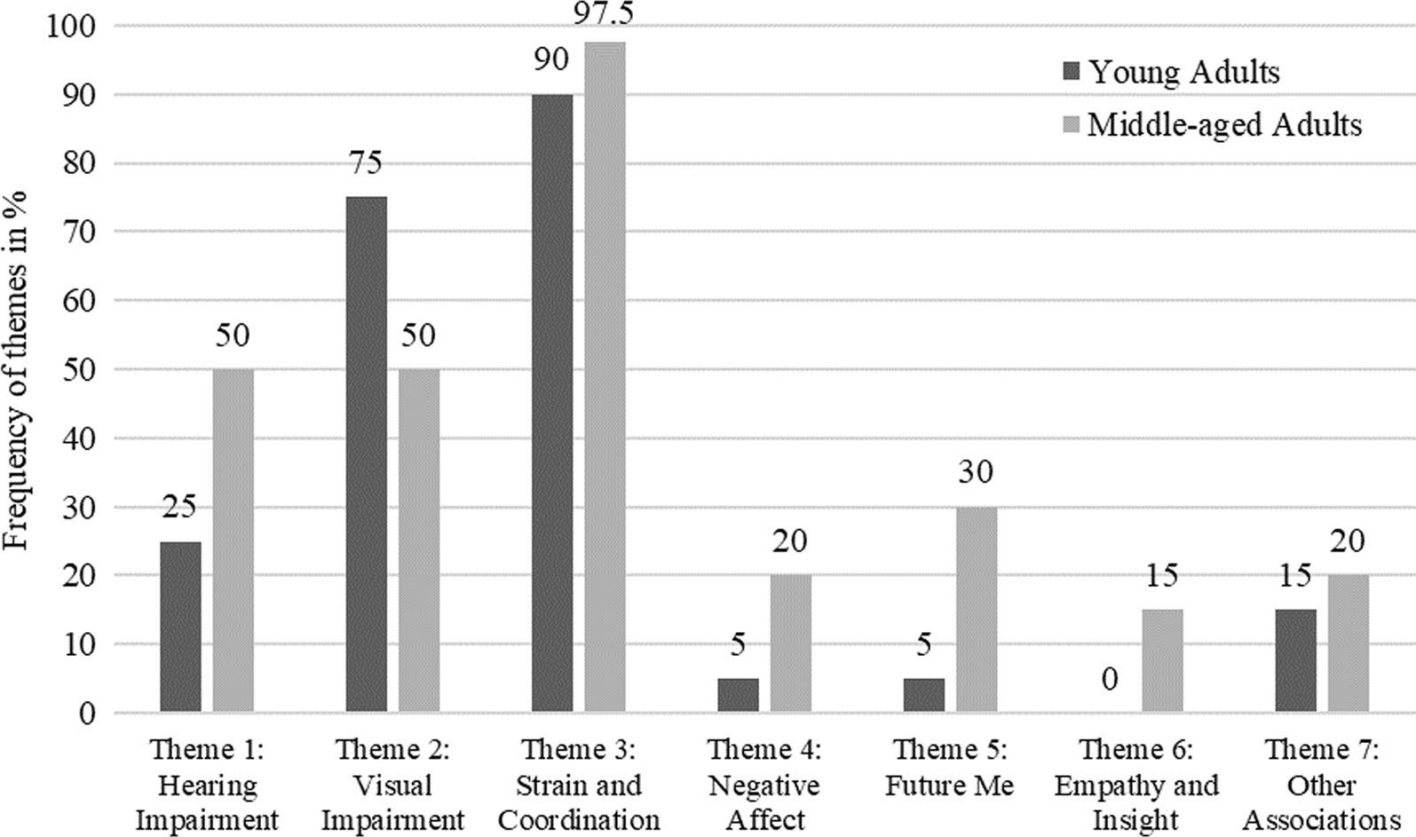
Frequency of themes among young and middle-aged adults (study 2)

Frequencies of themes in study 2 are depicted in Fig. 2, separated for age groups. Overall, Themes 1, 2, and 3, which comprised negative physical effects of the suit, were dominant in the data, led by Theme 3 (strain and coordination) with 97.5% among middle-aged and 90% among young participants. Chi-square tests were calculated to explore associations between themes and age group; if expected cell frequencies were below 5, Fisher’s exact test is reported. Marginal differences were found for Theme 1, hearing, *χ*^2^(1) = 3.43, *p* = .064, *φ* = .239 and Theme 2, vision, *χ*^2^(1) = 3.43, *p* = .064, *φ* = − .239, with younger adults reporting hearing impairments less frequently, but vision impairments more frequently than middle-aged adults. Theme 5, future me, was significantly mentioned more frequently by middle-aged adults *χ*^2^(1) = 4.91, *p*_(Fisher)_ = .024, *φ* = .286, which was also marginally the case for Theme 6, empathy and insight *χ*^2^(1) = 3.33, *p*_(Fisher)_ = .077, *φ* = .236, which was *not mentioned at all* by young adults, but by 15% of middle-aged adults. For negative affect, although not statistically significant, descriptive results indicate 20% mentions among middle-aged versus only 5% among young adults.

## Discussion

Following a mixed-method approach, we investigated the effects of wearing an age simulation suit on both general and personal views on aging in an age-diverse sample (study 1) and explored spontaneous associations among young adults versus middle-aged adults (study 2).

### Effects on views on aging

In the standardized pre- to post-assessments of views on aging, significant negative changes in both general and personal views on aging were observed with small-to-medium-sized effects. More specifically, participants reported more negative and less positive views regarding old people as a group after wearing the suit versus before. With respect to personal views, participants analogously expected more age-related losses and less age-related gains in post-assessment compared to the pretest. The negative shift in general views on aging found in our study is partly contradicting research that found predominantly small positive effects on general attitudes toward older adults, as summarized in recent reviews (e.g., Gerhardy et al. 2022). This might be due to a number of differences between our study and previous research in the area. First, social desirability might have played a more important role in previous studies, as study goals were often explicitly declared (i.e., age simulation as part of workshops to improve care, gain insight or/or improve empathy). Second, previous studies mostly included selective samples, young health care professionals in particular, who might react differently compared to our more diverse samples including various professions and age groups. Third, there are measurement and design differences, as studies used quite diverse instruments (see Gerhardy et al. 2022). Fourth, note once again that more sophisticated designs produced no or even negative effects in the previous research (e.g., Cheng et al. 2020).

For personal views, our findings mirror the scarce previous findings of Henry et al. (2011) who found higher fear of losses after an aging game (without a standardized aging suit, but with single components, e.g., limiting vision or fine motor abilities) and Schmidt et al. (2022) who reported negative shifts in (personal) aging-related cognitions. Further, even if our study only provides insights into short-term effects, it suggests caution in using age simulation as an educational means, because negative effects on views on aging might be a relevant side effect with importance for the wearers’ everyday life planning. For example, more negative views of oneself as an older person predicted less preparation for age-related changes, e.g., in the domain of health, in cross-sectional and longitudinal studies (Kornadt et al. 2015; Park et al. 2019). The observed sex differences, with women reporting more loss-related views after the simulation (but not before) with a medium-sized effect, might be due to the standardized weights of the suit that pose a proportional higher burden in relation to body weight and strength for most women. However, there might also be a relation to what Susan Sontag (1972) coined the double standard of aging, suggesting that an aging woman is judged more harshly than an aging man, and that ageist norms thus are internalized differently. This has received empirical support, e.g., Clarke and Korotchenko (2011) found in their review that men are less concerned than women about their aging appearances or age-related changes in their bodies.

Moreover, our data indicate that participants with higher education experienced smaller negative shifts in personal losses. The stronger negative trajectory among less educated participants might be due to observing less privileged older adults with lower socio-economic status in their social environment, less differentiated pictures of older adults and the aging process, and/or lower self-efficacy or control-beliefs, that have been associated with more negative future self-views (Park et al. 2019).

### Interpretation of qualitative results

Comparing the directed open question in study 1 (that asked to name the most burdensome features) with the undirected open format in study 2 yielded some analogous results with respect to impairments in hearing, vision, and strain/coordination that were also mentioned frequently in the spontaneous associations. However, study 2 provided more in-depth findings on psychological effects of the suit, namely negative affect, future me, and empathy/insight. Middle-aged adults reported significantly more future-related thoughts and (marginally) more empathy and insight toward older people than younger adults. In other words, middle-aged to young-old adults were more concerned with their emotions, future-self and older people than young adults who focused mainly on physical aspects. For example, for some middle-aged adults the change of perspective driven by the suit resulted in negative emotions because they imagined that their older loved ones face very similar struggles in daily life as they did when wearing the suit, and, additionally, the first-hand experience helped some middle-aged participants to critically reflect on their day-to-day perception and expectations of older people.

Middle-aged to young-old adults find themselves in a stage of life where they experience first aging-related changes and additionally, see their parents or other family members age, and might even act as caregivers to them. They might have accompanied relatives through their last years, therefore directly observing changes and declines in late stages of a person’s life. As a consequence of these first-hand aging experiences, the perhaps closer relationship to aging relatives, and observations of old age and death, there might have been a tendency of middle-aged and young-old adults to mention negative psychological effects, future-self related thoughts, and empathy or insight more frequently than young adults. Statements concerning their future-self or other older people were predominantly negative, e.g., worrying about their own future or pitying older people.

If there is truth in this interpretation, it would indicate that the effect that the aging suit has on middle-aged adults might be more intense and also more diverse than the effect on young adults. These findings are in line with previous literature that indicates an increased sensitivity of middle-aged adults to age-related changes and age stereotypes. As argued by Miche et al. (2014) experiencing age-related changes might be seen as more normative as people age, hence leading to greater sensitivity. This might in turn lead to more negative perceptions of one’s own aging. In accordance with stereotype embodiment theory by Levy (2009), study results by Kornadt et al. (2017) suggest that negative age stereotypes become internalized over time and a part of a person’s self-concept and that internalization is particularly strong among middle-aged adults.

In sum, our study might be seen as support for those who have an ambivalent attitude toward using an age simulation suit. For younger adults, particularly those aiming to become care professionals, positive effects in domains such as empathy and gaining a better internal view of what aging means cannot be seen as isolated from negative shifts in general and personal age views. Given that young adults are still far from old age, this might not be evaluated as a major issue. In some contrast, the effects in middle-ages adults seem emotionally more intensive than in younger adults and mid-age is a life period with heightened sensitivity to incorporating negative age views.

### Strengths and limitations

The present work contributed to existing literature in several ways. First, it is, to the best of our knowledge, the first study examining both general and personal views on aging within standardized anonymous pre/post-questionnaires, and additionally, qualitative data through open interviews and subsequent thematic analysis. Furthermore, the studies were not advertised as interventions to gain insight or empathy but foremost as a self-experience (study 1) and a motion study (study 2) which might have prevented biased answers alongside reducing the risk of social desirability. Consequently, the interview question in study 2 was undirected, leaving room to report any association that came to mind. Moreover, an age-diverse sample (study 1) and two age groups (study 2) were recruited, to overcome the bias of previous samples of young healthcare professionals. However, several limitations need to be discussed. Due to our non-probability sampling, e.g., people attending health fairs, open door days, or university classes, generalizability needs to be critically discussed. Our findings might predominantly concern individuals interested in research and health issues, with mostly higher education. In study 1, a passive control group without the aging suit but with analogous pre- and post-tests would have been desirable. In study 2, the smaller sample size posed an issue when conducting chi-squared tests, leading to interpretational limits. The sample of *N* = 60 met the estimated sample size for detecting medium-to-large effects but was not sufficient to detect small-to-medium-sized effects. Hence, a larger sample size might have led to more significant than (partly) marginal effects and would have allowed a more detailed description of the data. Moreover, the themes and counts alone do not reflect to which extent participants talked about certain aspects of the simulation. Considering data coding, there were some interpretational issues due to ambiguous statements, foremost due to the German “man” phrasing (here translated with a general “you”). “Man” describes a general person but can be used as a reference to oneself. Consequently, it was, at times, difficult to distinguish whether participants were really talking about themselves or any general person.

### Implications and outlook

Although aging suits are increasingly promoted to improve empathy or attitudes toward older adults, research on psychological effects as the current one suggests that some ambivalence is in place. To prevent or counteract possible negative effects, carefully debriefing participants and setting study experiences in perspective to what we know on age stereotypes and their negative effects seem necessary. As the effects in middle-ages adults seem emotionally more intensive than in younger adults and mid-age is a life period with heightened sensitivity to incorporating negative age views, aging suit interventions as a large-scale educative means may better focus on younger adults. Still, in mid-aged adults and potentially family caregivers it might also serve as a starting point for prevention (i.e., starting or maintaining an active lifestyle) or for interventions such as proactive home modification for aging in place.

## Data Availability

The datasets generated during and/or analyzed during the current study are available from the corresponding author on reasonable request.
